# Increased Abundance of Proteins Involved in Resistance to Oxidative and Nitrosative Stress at the Last Stages of Growth and Development of *Leishmania amazonensis* Promastigotes Revealed by Proteome Analysis

**DOI:** 10.1371/journal.pone.0164344

**Published:** 2016-10-24

**Authors:** Pedro J. Alcolea, Ana Alonso, Francisco García-Tabares, María C. Mena, Sergio Ciordia, Vicente Larraga

**Affiliations:** 1 Departamento de Microbiología Molecular y Biología de las Infecciones y Servicio de Proteómica y Genómica, Centro de Investigaciones Biológicas (Consejo Superior de Investigaciones Científicas), Calle Ramiro de Maeztu 9, 28040, Madrid, Spain; 2 Unidad de Proteómica, Centro Nacional de Biotecnología (Consejo Superior de Investigaciones Científicas), Calle Darwin 3, 28049, Madrid, Spain; INRS - Institut Armand Frappier, CANADA

## Abstract

*Leishmania amazonensis* is one of the major etiological agents of the neglected, stigmatizing disease termed american cutaneous leishmaniasis (ACL). ACL is a zoonosis and rodents are the main reservoirs. Most cases of ACL are reported in Brazil, Bolivia, Colombia and Peru. The biological cycle of the parasite is digenetic because sand fly vectors transmit the motile promastigote stage to the mammalian host dermis during blood meal intakes. The amastigote stage survives within phagocytes of the mammalian host. The purpose of this study is detection and identification of changes in protein abundance by 2DE/MALDI-TOF/TOF at the main growth phases of *L*. *amazonensis* promastigotes in axenic culture and the differentiation process that takes place simultaneously. The average number of proteins detected per gel is 202 and the non-redundant cumulative number is 339. Of those, 63 are differentially abundant throughout growth and simultaneous differentiation of *L*. *amazonensis* promastigotes. The main finding is that certain proteins involved in resistance to nitrosative and oxidative stress are more abundant at the last stages of growth and differentiation of cultured *L*. *amazonensis* promastigotes. These proteins are the arginase, a light variant of the tryparedoxin peroxidase, the iron superoxide dismutase, the regulatory subunit of the protein kinase A and a light HSP70 variant. These data taken together with the decrease of the stress-inducible protein 1 levels are additional evidence supporting the previously described pre-adaptative hypothesis, which consists of preparation in advance towards the amastigote stage.

## Introduction

American cutaneous leishmaniasis (ACL) is caused by 15 species of the genus *Leishmania* (Kinetoplastida: Trypanosomatidae), which includes species from the *Leishmania* and the *Viannia* subgenera. The latter is also able to cause mucocutaneous (MCL) leishmaniasis. *L*. *(L*.*) amazonensis* is one of the major etiological agents of ACL and it is grouped into the "*L*. *mexicana* complex". The initial ACL lesions are crater-shaped and they become ulcerated in the center with the progression of the disease. The species *L*. *mexicana*, *L*. *pifanoi* and *L*. *amazonensis* are also able to cause an infrequent form of the disease termed anergic diffuse cutaneous leishmaniasis (ADCL) [1], which is characterized by diffuse nodular lesions and total or partial anergy. ACL and ADCL are not life threatening, as a difference with visceral leishmaniasis (VL) caused by other species, but they are responsible for considerable morbidity.

According to the WHO epidemiological records, about 1.5 millon cases of CL are registered worldwide anually. Most ACL cases are concentrated in Brazil, Bolivia, Colombia and Peru [2, 3]. A recent increase in the cases of leishmaniasis has been observed globally, which may be explained by many interrelated factors as scarce control, poverty, poor hygiene, migrations, return to rural areas, penetration into the jungle, changes in ambient humidity, deforestation, etc. ACL is a zoonosis and the major reservoirs are rodents when the etiological agents belong to the *L*. *mexicana* complex [2].

The life cycle of the parasite involves an invertebrate host that injects the motile promastigote stage of the parasite to the mammalian host when feeding from venules of the dermis. The vector belongs to the genus *Lutzomyia* (Psychodidae: Phlebotominae) in the case of New World species of the parasite like *L*. *amazonensis*. Promastigotes are engulfed by host phagocytes and differentiate to amastigotes. When a sand fly feeds from blood of an infected mammal, amastigotes are released from phagocytes, transform into undifferentiated (procyclic) promastigotes and begin a developmental process from the procyclic to the more infective metacyclic stage.

A remarkable number of trypanosomatid genome sequences were assembled and annotated [4–6], which allowed for studying differential gene expression in different species, thus contributing to the knowledge about the biology of the parasite [7]. This is essential for future discovery of drug targets and vaccine candidates. In the case of *L*. *amazonensis*, two-dimension electrophoresis (2DE) maps of promastigotes were described [8, 9]. In another study, the effect of culture passage and the resulting decrease of infectivity in the proteome profile of stationary phase promastigotes were evaluated by the same approach [10]. Also, a transcriptome analysis focused in the search of genes associated to antimony resistance was performed in promastigotes at the mid-logarithmic phase of growth in axenic culture [11]. However, differential gene expression throughout growth and development of the promastigote stage of *L*. *amazonensis* in culture and within the sand fly gut has not been still explored. The aim of this study is comparing different time points of growth of promastigotes in culture by 2DE and subsequent matrix-assisted laser desorption-ionization tandem time-of-flight mass spectrometry (MALDI-TOF/TOF).

## Materials and Methods

### Parasites

Promastigotes of the *L*. *amazonensis* strain MHOM/Br/79/María were kindly provided by Alfredo Toraño and Mercedes Domínguez [12] and cultured at 27°C, pH 7.2 in liquid media composed of RPMI 1640 supplemented with L-glutamine (Life Technologies, Carlsbad, CA), 10% heat inactivated fetal bovine serum (Lonza, Basel, Switzerland) and 100 μg/ml streptomycin– 100 IU/ml penicillin (Life Technologies). The initial cell density was adjusted to 2 x 10^6^ cells/ml in all cultures and growth was registered daily at the light microscope using a Neubauer chamber. Culture samples containing 10^8^ promastigotes were obtained at the early-logarithmic, mid-logarithmic, late-logarithmic and stationary phases.

### Total protein extracts

Promastigotes (10^8^ per sample) were harvested at 2,000 g for 10 min and washed once in PBS. Then, the sediment was carefully resuspended in 150 μl lysis buffer (8.4 M urea, 2.4 M thiourea, 5% CHAPS, 50 mM DTT, 1% Triton X-100, 50 μg/ml DNase and Mini EDTA-free Protease Inhibitor Cocktail according to the manufacturer’s instructions –Roche, Mannheim, Germany) and immediately mixed by mild rotation at 4°C for 30 min. Then, the protein extracts were centrifuged at 8,000g for 10 min. The supernatant was recovered, precipitated with methanol/chloroform [13], dried at room temperature for 5 min and resuspended in 2X rehydration buffer (7 M urea, 2 M thiourea, 4% CHAPS, 0.003% bromophenol blue). Quantification was performed with the RC DC protein assay kit (BioRad) following the manufacturer’s instructions and the results were compared with SDS-PAGE results as described [14].

### Two-dimensional electrophoresis (2DE)

Fifty μg of total protein per sample were diluted to a final volume of 140 μl in 2X isoelectrofocusing (IEF) buffer (18.2 M DTT and 0.5% *IPG buffer solution* pH 3–10, BioRad). Then, samples were subject to IEF on 7 cm IPG pH 3–10 non-linear gradient strips (BioRad) in a Protean IEF Cell system (BioRad) following the manufacturer's instructions. Each run comprised seven steps (50 V for 12h, 250 V for 1h, 500 V for 1h, 1000 V for 1h, 2000 V for 1h, linear ramp to 8000 V for 1h and 8000 V up to 3500 V·h), reaching more than 12,000 V·h in every one. Then, the strips were run by 12% SDS-PAGE in a pre-cooled MiniProtean 3 Dodeca Cell system (BioRad) at 0.5 W/gel for 30 min and then at 1.5 W. The run was stopped 5 min after the die-front reached the bottom edge. The gels were stained with SYPRO Ruby (BioRad) and the gel images were acquired with EXQuest Spot Cutter (BioRad) according to the manufacturer's instructions.

The gel images were processed and analyzed with PDQuest 2D Advanced 8.0.1 software (BioRad) according to the manufacturer's instructions. First, the images were cropped and spots were detected automatically with the Spot Detection Parameter Wizard at a sensitivity value of 3.5. Then, all detected spots were manually checked by observation of 3D density graphs, single spot quantitation histograms and 2DE gel images. Normalization was performed by the Total Quantity in Valid Spots algorithm. Normality was checked by the Kolmogorov-Smirnov test and statistical inference of differential protein abundance was performed by the Student's t-test at the 0.05 significance level. Reproducibility was assured by preparing three independent biological replicate samples. However, all 2DE runs were performed simultaneously, which is essential for technical reproducibility across gels. For this purpose, all samples were stored at -80°C until use.

### Protein identification by MALDI-TOF/TOF

After excision of the selected spots with EXQuest Spot Cutter (BioRad), they were digested with trypsin and prepared for MALDI-TOF/TOF mass-spectrometry as described [14]. The digests were deposited in OptiTOF^™^ Plates (Life Technologies). Each well contained a 0.8 μl drop of peptides mixed with 0.8 μl of 3 μg/μl α-cyano-4-hydroxycinnamic acid (Sigma). The mixtures were allowed to dry at room temperature and run in an ABI 4800 MALDI-TOF/TOF mass spectrometer (Life Technologies) in positive reflector mode at 25 kV for MS and 1 kV for MS/MS. The data obtained were analyzed with ABI 4000 Series Explorer software 3.6 (Life Technologies). Peptide mass fingerprinting (PMF) and MS/MS spectra of fragment ions were smoothed and corrected to the zero baseline using routines embedded in ABI 4000 Series Explorer Software v3.6. Each PMF spectrum was internally calibrated with the mass signals of trypsin autolysis ions to reach a typical mass measurement accuracy < 25 ppm. Known trypsin and keratin mass signals, as well as potential sodium and potassium adducts (+21 Da and + 39 Da) were removed from the peak list. Protein identifications were performed with MASCOT 2.1 using Global Protein Server Explorer 4.9 (Life Technologies). The following search parameters were introduced: enzyme, trypsin; allowed missed cleavages, 1; carbamidomethyl cystein as fixed modification by treatment with iodoacetamide; variable modifications, oxidation of methionine; mass tolerance was set to ± 50 ppm for precursors and to ± 0.3 Da for MS/MS fragment ions. The confidence interval for protein identification was set to ≥ 95% (p < 0.05) and only peptides with an individual ion score above the identity threshold (52) were considered correctly identified. The reference template for MASCOT identifications was the genome sequence of *L*. *mexicana* (http://tritrypdb.org/common/downloads/release-9.0/LmexicanaMHOMGT2001U1103/fasta/data/) because the genome of *L*. *amazonensis* has not been sequenced yet and this species is included in the "*L*. *mexicana* complex". All data were also run against against individual *L*. *amazonensis* sequences within the NCBInr database. The identifications are available in the PRIDE repository of the ProteomeXchange Consortium [15] with the accesion number PXD002939.

### Evaluation of resistance to nitric oxide (NO) by the [3'-(4,5-dimethylthiazol-2-yl)-2,5-diphenyltetrazolium bromide] (MTT) assay

Logarithmic and stationary phase promastigotes were washed with PBS and at 26°C for 4 h at a cell density of 10^6^ in a final volume of 100 μl in the presence of different concentrations (1:2 serial dilutions from 16 to 0.25 mM) of NaNO_2_ (NO donor) in PBS pH 5.0. Next, suspensions were centrifuged at 700g for 10 min and the sediments were resuspended in 100 μl PBS pH 7.0. Then, 10 μl of 5 mg/ml MTT were added and the cells were incubated at 25°C for 4 h. The reaction was stopped and formazan solubilized by adding 100 μl of 0.4N HCl in 50% isopropanol. Finally, A_490nm_ was measured to assess formazan accumulation, which is related to viability [16]. The differences were statistically assessed with the paired Student’s t-test from three biological replicates of the experiment.

## Results and Discussion

### 2DE-MS/MS analysis of *L*. *amazonensis* promastigotes

**Fig 1:**
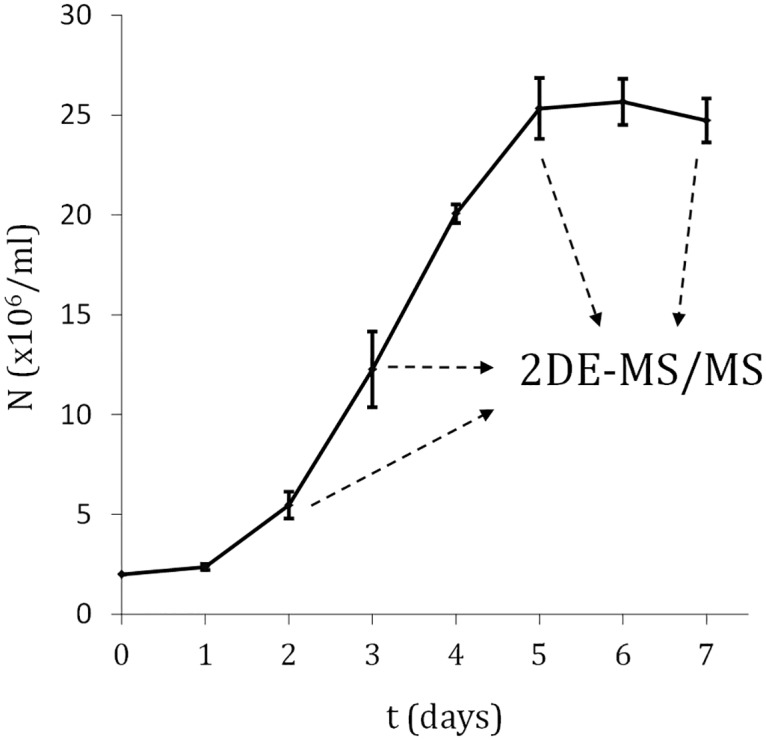
Growth curve of *L*. *amazonensis* promastigotes. Total protein samples were prepared and quantified at day 2 (early logarithmic phase), day 3 (mid logarithmic phase), day 5 (late logarithmic phase/early stationary phase) and day 7 (stationary phase).

**Fig 2:**
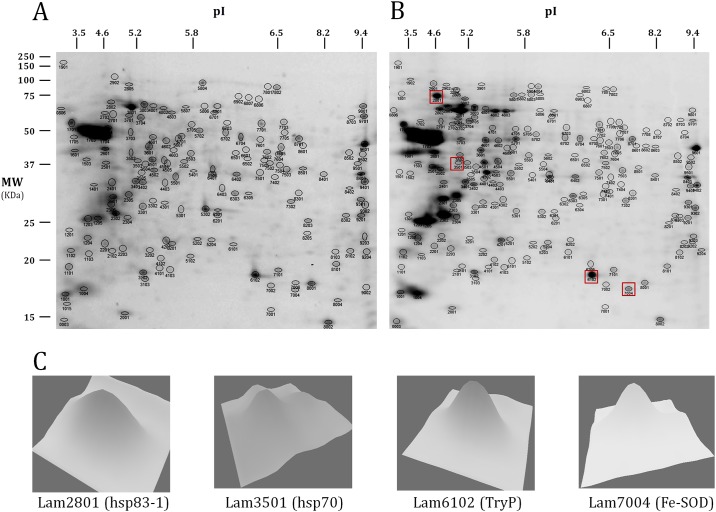
Examples of 2DE of total protein extracts of *L*. *amazonensis* promastigotes. (A) Early logarithmic (day 2) and (B) stationary phase (day 7). IEF was performed in a non-linear 3–10 pH interval. (C) 3D density graphs of spots Lam2801, Lam3501, Lam6102 and Lam7004 obtained with PD Quest software.

Total protein was extracted from *L*. *amazonensis* axenic cultures of promastigotes at early-logarithmic (day 2), mid-logarithmic (day 3), late-logarithmic (day 5) and stationary (day 7) phase (Fig 1). Average protein concentrations (mg/ml) were: 4.4 ± 0.2 (day 2 promastigotes); 4.5 ± 0.4 (day 3); 4.4 ± 0.1 (day 5); 4.5 ± 0.2 (day 7). (Fig 2). The spots were automatically detected with the Spot Detection Parameter Wizard. Sensitivity was adjusted to 3.5 in order to filter out the non-identifiable faintest spots according to experimental setup with the mass spectrometer. An average of 202 spots were detected per gel and the cumulative non-redundant number is 339 (S1 Table). The cut-off ratio values were 1.7 for up-regulation and 0.6 for down-regulation (1.7 and -1.7 fold changes), respectively (S1 Fig, S1 Table). Statistical significance was inferred with the Student’s t test (p < 0.05) because the outcome of the Kolmogorov-Smirnov test was p = 0.318, 0.280 and 0.273 for the comparisons day3:day2, day5:day2 and day7:day2, respectively. Ratios are relative to early-logarithmic phase promastigotes (day 2). Sixty three differentially expressed proteins out of 339 could be identified by MALDI-TOF/TOF (Fig 2, Table 1, S1 Table) against the TriTrypDB *L*. *mexicana* genome annotations. All selected proteins were identified and the MASCOT score values were significant, except for one spot that could not be identified with the *L*. *mexicana* reference genome sequence but the NCBInr database instead (Table 2, spot Lam1601, soluble promastigote surface antigen fragment PSA-31S, part of the PSA-38S). In fact, this *L*. *amazonensis* gene had been previously characterized [17]. Random identification of constitutively expressed proteins was also performed (Table 2), which occasionally allowed for retrieving additional information about relative expression in different pathways (see below). In certain cases, different spots contain the same protein, which may be due to protein processing, post-translational modifications or protein aggregation. In the next sections, this has been cited as "protein variant". In principle, the nature of the variations is unknown but the 2DE-resolved variants could be separately analyzed and identified. The comparison of experimental and/or predicted molecular weight and isoelectric point provides insight (Table 3). For example, three variants of the protein LmxM.15.1160 were found. Two of them present a similar MW but differ in the estimated pI value in 0.5 pH units, whereas the MW of the third one is about 40% lower and the pI 8.5, which strongly suggests that a considerable portion of the protein sequence has been removed during intracellular protein processing. The most striking changes in experimental MW and pI (Table 3) are discussed below according to changes in relative abundance of variants of the same protein.

**Table 1:** Differential gene expression regulation in *L*. *amazonensis* promastigotes. The MW and pI values provided were estimated by the 2DE analysis software PD Quest. Theoretical values can be obtained from the *L*. *mexicana* LmxM genome databank within TriTrypDB by introducing the gene Ids. provided in this table.

Spot	TriTrypDB ID	Protein	MASCOT score (p < 0.05)	MW (KDa)	pI	Day3:Day2 (p < 0.05)	Day5:Day2 (p < 0.05)	Day7:Day2 (p < 0.05)
Lam 102	LmxM.34.4470	Hypothetical protein, conserved	300	23.74	3.3	0.51 (-1.96)	0.39 (-2.56)	0.56 (-1.78)
Lam1101	LmxM.04.0770/ 60	Unspecified product	96/89	23.16	4.0		0.5 (-2.00)	0.31 (-3.22)
Lam1103	LmxM.14.0190	Hypothetical protein, conserved	512	25.18	4.7	0.57 (-1.75)		
Lam1204	LmxM.36.3210	14-3-3 protein 1, putative	442	27.66	4.6		0.36 (-2.78)	
Lam1205	LmxM.08.1230	Beta tubulin	640	30.55	4.8			2.55
Lam1503	LmxM.25.0750	Protein phosphatase, putative	512	44.71	4.6	0.53 (-1.89)		0.56 (-1.78)
Lam1702	LmxM.13.0160	Protein kinase A regulatory subunit, putative	70	66.63	4.7			24.28
Lam2001	LmxM.15.0281/75	Ribonucleoprotein p18, mitochondrial precursor, putative	247/247	17.44	5.2		0.34 (-2.94)	
Lam2202	LmxM.13.0300	Unspecified product	206	27.99	5.1			40.22
Lam2304	LmxM.14.0310	Proteasome alpha 3 subunit	187	31.14	5.3			1.86
Lam2401	LmxM.25.1710	Pyruvate dehydrogenase E1 beta subunit, putative	133	38.28	5.2	0.27 (-3.7)	0.17 (-5.89)	
Lam2801	LmxM.32.0312/ 14/16	Heat shock protein 83–1	614/614/614	91.86	5.0	3.03		
Lam3102	LmxM.23.0040	Peroxidoxin	589	22.38	5.5		0.36 (-2.78)	
Lam3201	LmxM.34.1480	Arginase (ARG)	67	27.65	5.5		18.08	28.89
Lam3301	LmxM.34.1540	Rieske iron-sulfur protein, mitochondrial precursor, putative (RISP)	105	32.87	5.5		0.38 (-2.63)	
Lam3501	LmxM.28.2770/80	Heat-shock protein hsp70, putative	234/165	44.50	5.4			24.06
Lam3505	LmxM.07.0640	Hypothetical protein, conserved	320	40.57	5.5		0.09 (-11.1)	
Lam3601	LmxM.14.1160	Enolase	586	49.17	5.6		0.48 (-2.08)	0.49 (-2.04)
Lam3701	LmxM.36.2020/30	Chaperonin hsp60, mitochondrial precursor	820/356	67.83	5.3		0.01 (-100)	
Lam3704	LmxM.36.2030/20	Chaperonin hsp60 mitochondrial precursor	700	66.34	5.5		0.21 (-4.76)	0.49 (-2.04)
Lam3705	LmxM.36.6650	2,3-bisphosphoglycerate-independent phosphoglycerate mutase	304	68.99	5.5		60.01	
Lam3801	LmxM.28.2770/80	Heat-shock protein hsp70, putative	990/211	73.81	5.4		0.44 (-2.27)	
Lam3803	LmxM.29.2490/60/70	Heat shock 70-related protein 1, mitochondrial precursor, putative	874/727/726	76.87	5.5		0.32 (-3.12)	
Lam3901	LmxM.22.1540	Alanyl-tRNA synthetase, putative	376	112.48	5.6	1.83		
Lam4001	LmxM.23.0200	Endoribonuclease L-PSP (pb5), putative	360	12.93	5.7			1.76
Lam4002	LmxM.34.1300	Ubiquitin-conjugating enzyme E2, putative	135	12.16	5.5		0.46 (-2.17)	
Lam4401	LmxM.23.0110	Mannose-1-phosphate guanyltransferase (GDP-MP)	344	39.32	5.6		2.63	
Lam4403	LmxM.30.2250	3,2-trans-enoyl-CoA isomerase, mitochondrial precursor, putative	251	37.59	5.7	0.59 (-1.70)		
Lam5001	LmxM.15.1160/40	Tryparedoxin peroxidase	200/75	12.88	5.9			30.53
Lam5002	LmxM.20.1280	Calpain-like cysteine peptidase, Clan CA, family C2, putative	324	13.92	6.1		0.02 (-50.00)	0.01 (-100.00)
Lam5103	LmxM.15.1160/40	Tryparedoxin peroxidase	216/82	21.97	6.0	47.02		
Lam5302	LmxM.28.2740	Activated protein kinase C receptor (LACK)	510	32.08	6.1	0.51 (-1.96)	0.27 (-3.70)	0.25 (-4)
Lam5501	LmxM.06.0370	Glutamine synthetase, putative	262	41.01	5.9		0.38 (-2.63)	0.54 (-1.85)
Lam5502	LmxM.32.2300	UDP-glucose-4'-epimerase, putative	354	43.91	5.9		0.55 (-1.82)	
Lam5504	LmxM.06.0880	Acyl-coenzyme A dehydrogenase, putative	146	44.02	6.1			14.91
Lam5801	LmxM.30.0010	5-methyltetrahydropteroyltriglutamate-homocysteine methyltransferase, putative (MET6)	575	92.78	5.9	3.49		
Lam5804	LmxM.36.0180	Elongation factor 2	560	107.45	6.1	1.76	0.42 (-2.38)	0.41 (-2.43)
Lam5901	LmxM.36.0180	Elongation factor 2	449	107.63	6.0	2.35		
Lam6102	LmxM.15.1160/40	Tryparedoxin peroxidase	305/163	21.94	6.5	0.58 (-1.72)		
Lam6403	LmxM.36.4170	Oxidoreductase, putative	194	36.13	6.3		0.03 (-33.3)	
Lam6701	LmxM.34.3860	T-complex protein 1, eta subunit, putative	402	69.50	6.2		0.06(-16.67)	
Lam6702	LmxM.36.2660	Dihydrolipoamide acetyltransferase	128	55.74	6.3		0.45 (-2.22)	
Lam6703	LmxM.11.0630/20	Aminopeptidase, putative,metallo-peptidase, Clan MF, Family M17/aminopeptidase, putative	325/186	61.94	6.3	3.33		2.72
Lam6704	LmxM.31.3310	Dihydrolipoamide dehydrogenase	565	55.19	6.4			0.57 (-1.75)
Lam6801	LmxM.28.2770	Heat-shock protein hsp70, putative	190	73.71	6.2		0.31 (-3.22)	
Lam6806	LmxM.31.2951/50	Nucleoside diphosphate kinase b	553/553	88.79	6.8	0.09 (-11.1)		0.06 (-16.67)
Lam7003	LmxM.31.2951/50	Nucleoside diphosphate kinase b	544/544	13.19	7.5		0.31 (-3.22)	
Lam7004	LmxM.31.1820/30	Iron superoxide dismutase, putative	476/265	19.91	7.6	1.75		
Lam7603	LmxM.34.1380	Mitochondrial processing peptidase, beta subunit, putative, metallo-peptidase, Clan ME, family M16	522	52.84	7.0	40.36		
Lam7605	LmxM.34.1380	Mitochondrial processing peptidase, beta subunit, putative, metallo-peptidase, Clan ME, family M16	681	50.37	7.5	1.76		
Lam7701	LmxM.34.3860	T-complex protein 1, eta subunit, putative	427	63.99	6.8		0.4 (-2.5)	0.52 (-1.92)
Lam7702	LmxM.36.0180	Elongation factor 2	300	60.30	6.9	3.34		
Lam7703	LmxM.36.0070	Stress-inducible protein STI1 homolog	318	65.49	7.4	0.5 (-2.00)	0.24 (-4.17)	0.59 (-1.70)
Lam7801	LmxM.18.0510	Aconitase, putative	502	103.24	7.0		0.43 (-2.32)	
Lam8003	LmxM.06.0120	Cyclophilin	321	13.18	9.2	0.51 (-1.96)	0.48 (-2.08)	
Lam8005	LmxM.26.1380	Prefoldin-like protein/Cyclophilin, putative	125/106	21.02	9.5	30.56		
Lam8101	LmxM.24.0850	Triose phosphate isomerase	621	23.26	9.1	0.44 (-2.27)	0.43 (-2.32)	
Lam8102	LmxM.36.0070	Stress-inducible protein STI1 homolog	347	25.49	9.4		0.43 (-2.32)	
Lam8201	LmxM.24.1980	Hypothetical protein, conserved	404	30.12	9.2			15.15
Lam8501	LmxM.31.0840	Hypothetical protein, conserved	188	42.92	8.2		0.36 (-2.78)	0.33 (-3.03)
Lam8701	LmxM.25.1120	Aldehyde dehydrogenase, mitochondrial precursor	497	54.99	8.0			1.77
Lam8703	LmxM.07.0340	ATP-dependent DEAD/H RNA helicase, putative	201	67.09	9.4	6.04		
Lam8706	LmxM.03.0200/24.0770	Delta1-pyrroline-5-carboxylate dehydrogenase, putative/Malic enzyme, putative	181/163	59.28	9.1	20.4		

**Table 2:** Constitutively expressed proteins in *L*. *amazonensis* promastigotes. The MW and pI values provided were estimated by the 2DE analysis software PD Quest. Theoretical values can be obtained from the *L*. *mexicana* LmxM genome databank within TriTrypDB by introducing the gene Ids. provided in this table.

Spot	TriTrypDB ID	Protein	MASCOT score (p < 0.05)	MW (KDa)	pI
Lam1601	ACY70938 (NCBI Id.)	Soluble promastigote surface antigen PSA-31S	221	74.38	4.4
Lam2502	LmxM.32.0312/14/16	Heat shock protein 83–1	288/288/288	61.34	5.1
Lam2601	LmxM.36.6940	Protein disulfide isomerase	395	73.85	5.5
Lam2803	LmxM.28.1200	Glucose-regulated protein 78, putative	611	105.47	5.2
Lam2804	LmxM.36.1370	Transitional endoplasmic reticulum ATPase, putative, valosin-containing protein homolog	185	147.73	5.7
Lam3001	LmxM.08_29.1160	Tryparedoxin 1, putative (TXN1)	228	13.20	5.4
Lam3802	LmxM.29.1760/50	Paraflagellar rod protein 1D, putative	312/312	127.12	5.4
Lam4601	LmxM.32.2540	Carboxypeptidase, putative,metallo-peptidase, Clan MA(E), Family M32	457	79.14	5.7
Lam4801	LmxM.26.1570	Thimet oligopeptidase, putative,metallo-peptidase, Clan MA(E), Family M3	247	98.15	5.7
Lam4803	LmxM.28.2770/20	Heat-shock protein HSP70, putative	199	97.64	5.8
Lam5503	LmxM.01.0770	Unspecified product	315	73.92	6.0
Lam5506	LmxM.36.2950	Succinyl-CoA ligase (GDP-forming) beta chain, putative	748	54.02	6.2
Lam5702	LmxM.27.1260	T-complex protein 1, beta subunit, putative	279	80.04	6.1
Lam5803	LmxM.36.0180	Elongation factor 2	136	124.23	6.0
Lam6402	LmxM.25.1610	Hypothetical protein, conserved	79	62.25	6.4
Lam6705	LmxM.27.1220	Hypothetical protein, conserved	145	78.46	6.6
Lam7503	LmxM.10.0290	Isocitrate dehydrogenase (NADP), mitochondrial precursor, putative	283	65.21	7.5
Lam7602	LmxM.18.0670	Citrate synthase, putative	273/270	74.91	6.8
Lam7604	LmxM.21.0340	Mitochondrial processing peptidase alpha subunit, putative,metallo-peptidase, Clan ME, Family M16	223	74.26	7.2
Lam8001	LmxM.15.1160/40	Tryparedoxin peroxidase	248/102	22.46	8.5
Lam8103	LmxM.21.0850	Xanthine phosphoribosyltransferase	319	24.12	9.4
Lam8203	LmxM.36.5120/10	40S ribosomal protein SA, putative	354/255	34.03	9.4
Lam8403	LmxM.36.1260	Fructose-1,6-bisphosphate aldolase	458	66.15	9.5
Lam8601	LmxM.32.2340	Succinyl-CoA:3-ketoacid coenzyme A transferase, mitochondrial precursor, putative	712	68.41	8.2
Lam9201	LmxM.02.0460	Voltage-dependent anion-selective channel, putative (VDAC)	298	36.34	9.5
Lam9301	LmxM.25.2130/40	Succinyl-CoA synthetase alpha subunit, putative	319/319	47.70	9.5
Lam9401	LmxM.29.2980	Glyceraldehyde-3-phosphate dehydrogenase, glycosomal	458	48.53	9.5

**Table 3:** Remarkable examples of protein variants. Comparison of experimental (Exp) and predicted theoretical (P) MW and pI.

TriTryp Id.	Protein	^Exp^MW	^P^MW	^Exp^pI	^P^pI
LmxM.15.1160	TryP	12.9	22.2	8.5	6.7
LmxM.15.1160	TryP	22.0	22.2	6.0	6.7
LmxM.15.1160	TryP	21.9	22.2	6.5	6.7
LmxM.28.2770	HSP70	44.5	71.2	5.4	5.2
LmxM.28.2770	HSP70	73.8	71.2	5.4	5.2
LmxM.28.2770	HSP70	73.7	71.2	6.2	5.2
LmxM.28.2770	HSP70	97.6	71.2	5.8	5.2
LmxM.29.2490	HSP70-rel1	76.9	72.5	5.5	5.7
LmxM.31.2951	NDKb	88.79	16.7	6.8	7.5
LmxM.31.2951	NDKb	13.2	16.7	7.5	7.5
LmxM.34.1380	M16 peptidase	52.8	54.6	7.0	7.1
LmxM.34.1380	M16 peptidase	50.4	54.6	7.5	7.1
LmxM.36.0180	EF2	107.4	94.1	6.1	6.0
LmxM.36.0180	EF2	107.6	94.1	6.0	6.0
LmxM.36.0180	EF2	60.3	94.1	6.9	6.0

The differential gene expression results at the protein abundance level in the promastigote differentiation process studied herein are compared below with expression profiles reported in other species. Experimental variation between studies has been considered. In other cases, the information is complemented with reported data about the differentiation process of promastigotes to amastigotes.

### Differential protein abundance in *L*. *amazonensis* promastigotes

#### Regulation of gene expression

Proteins involved in translation and post-transcriptional regulation of gene expression, play a key role in the biology of trypanosomatids because these organisms lack most transcriptional regulation mechanisms (reviewed in [18]). The abundance of some of them changes in the growth and differentiation process of *L*. *amazonensis* promastigotes. First, three variants of the translation elongation factor 2 (EF2) are up-regulated in mid-logarithmic with respect to early-logarithmic phase promastigotes, as well as two different DEAD/DEAH RNA helicases and the alanine-tRNA synthetase (Table 1). One EF2 variant is considerably lighter and slightly more basic (60.3 KDa, pI 6.9) than the others (Table 3), which suggests that it is partially proteolyzed during processing. The two other variants may have been post-translationally modified, according to the differences in the theoretical MW values (Tables 1 and 3). A heavier variant of the EF2 is constitutively expressed (Tables 2 and 3). This constitutive variant may contain additional post-translational modifications, probably glycosylation regarding to the increase in MW (Table 3). The increase in abundance of the EF2 variants (Tables 1 and 2) suggests changes in translational regulation at an intermediate point of growth and differentiation. The EF2 was described to be an immunostimulatory protein in *L*. *donovani* [19], but the importance of this protein for differentiating promastigotes may rely more directly in regulation of translation rather than preparation in advance for colonization of the host cell within the mammalian host (see pre-adaptative hypothesis below). An alternative possibility could be the decrease of the steady-state levels in case the immune response elicited by the protein leads to progression of the disease.

Another protein involved in gene expression regulation is the endoribonuclease L-PSP (pb5), which was found to be down-regulated in amastigotes of species causative of cutaneous (*L*. *mexicana*, *L*. *major*) and visceral (*L*. *donovani*, *L*. *infantum*) leishmaniasis [20–24]. However, information about differential abundance of this protein throughout the promastigote growth and differentiation process is not available so far. In the case of *L*. *amazonensis*, this endoribonuclease is over-expressed in stationary phase promastigotes, therefore at the end of the differentiation process (Table 1).

#### Protein folding

The heat shock proteins HSP70 and HSP83-1 were described to be abundant and constitutive throughout the main stages of the life cycle of *Leishmania* spp. [25, 26]. Differential expression in the promastigote stage of *L*. *infantum* was not detected at the transcript and protein levels [14, 20]. In the case of *L*. *amazonensis*, distinct variants of both chaperones are constitutively or differentially expressed in promastigotes throughout growth and differentiation (Tables 1 and 2). Two HSP70 (LmxM.28.2770) variants of the same MW but different estimated pI (5.4 and 6.2) are less abundant at late-logarithmic phase (Tables 1 and 3). The HSP70-related protein 1 (HSP70-rel1), encoded by a different gene (LmxM29.2490), is slightly heavier (Table 3) and its relative abundance is analogous, whereas a lighter variant of the LmxM.28.2770 HSP70 (44.5 KDa) of estimated pI around the theoretical value (5.2) is highly up-regulated (24-fold) in stationary phase promastigotes (Tables 1 and 3). Finally, a heavier variant (97.6 KDa) is constitutive (Tables 2 and 3). Therefore, alternation of HSP70 variants originated by protein processing or post-translational modifications is observed at the last stages of the growth and differentiation process of *L*. *amazonensis* promastigotes. Simultaneously, the chaperonin HSP60 is down-regulated (late-logarithmic phase), whereas the differentially regulated variants of the HSP83 are down-regulated at mid-logarithmic phase, an earlier point of growth and differentiation. According to our results, the overall HSP70 levels increase in the ongoing of differentiation of promastigotes considering all variants together. As HSP70 proteins of *L*. *infantum* and *L*. *donovani* were described to be immunostimulatory [19, 27], they may be involved in colonization of the mammalian host phagocytesby differentiated promastigotes, though it may lead to susceptibility or resistance of the host depending on the type of immune response elicited.

According to previous data, the stress-inducible protein 1 homolog (STI1) is constitutively expressed in the developmental process of stationary phase promastigotes to amastigotes in the case of *L*. *infantum* [28] but no information is available about growth and development of promastigotes. In the case of *L*. *amazonensis*, the STI1 is down-regulated at late logarithmic phase (Table 1), simultaneously to some HSPs mentioned above.

Two variants of the η subunit of the T-complex protein 1 (TCP1η) are down-regulated in late logarithmic phase promastigotes (Table 1), whereas the TCP1β subunit is constitutively expressed (Table 2). Therefore, the down-regulation of the TCP1η, the STI1 and the HSPs mentioned above is simultaneous and takes place at one of the final stages of growth and differentiation of *L*. *amazonensis* promastigotes in culture. The exact function of the TCP complex is not known in *Leishmania* spp., although it has been suggested that the TCP1γ subunit may participate in maintaining the structural dynamics of the cytoskeleton [29]. Changes in cell morphology throughout promastigote differentiation and flagellar movement demand continuous re-organization of the cytoskeleton. Differential expression of the TCP1η has not been reported in *L*. *infantum* promastigotes differentiating in culture so far, although the encoding gene is up-regulated at the transcript level in metacyclic promastigotes isolated from the sand fly anterior midgut [30].

A cyclophilin variant is down-regulated in mid-logarithmic phase *L*. *amazonensis* promastigotes, whereas another variant is constitutively expressed. Cyclophilins are able to prevent aggregation of adenosine kinase domain-containing proteins [31, 32] like nucleoside diphosphate kinases (NDK). Two NDKb variants encoded by the same gene but very different in MW (presumably due to processing) are up-regulated at the beginning of growth and differentiation of promastigotes (early-logarithmic phase) (Tables 1 and 3). Up-regulation of the NDKb at the transcript level in procyclic *L*. *infantum* promastigotes [33] agrees with the profile found in *L*. *amazonensis*. The NDKs are involved in nucleotide biosynthetic processes, which may occur preferentially at the initial stages of growth and differentiation of promastigotes as suggested by the results. The 14-3-3 protein 1 relative abundance profile is the same as for the NDKb in *L*. *amazonensis* promastigotes. The exact functions of 14-3-3 proteins are unknown, although they have been related to many cellular processes. An intrinsic NDK activity has been associated to these proteins in general [34] and antiapoptotic properties of the *L*. *donovani* orthologs have been reported [35]. In fact, the 14-3-3 protein is able to prolong the lifespan of the infected host phagocyte. However, given the multiple functions of this protein, the implications of its increased levels at the beginning of promastigote growth and differentiation are not known so far. The decrease of Cph may cause an increase in aggregation of NDKb and 14-3-3, which may be a clue to explain their decrease at an earlier stage.

#### Proteolysis

Two components of the ubiquitin-proteasome system are differentially abundant throughout growth and differentiation of *L*. *amazonensis* promastigotes: the ubiquitin-conjugating enzyme E2 (UbqC-E2) is down-regulated in late-logarithmic phase, whereas the proteasome α-3 subunit is up-regulated in stationary phase promastigotes (Table 1). None of them have been detected in differential gene expression analysis during development of promastigotes of any *Leishmania* species in culture so far. However, it has been described that the the E2 steady-state transcript levels are higher in cultured promastigotes than in promastigotes obtained from the sand fly gut [30, 36]. The 14-3-3 protein is associated to other components of the proteasome [37]. As mentioned above, the abundance of this protein decreases at the last stages of growth and development of *L*. *amazonensis* promastigotes (late-logarithmic phase promastigotes), which is simultaneous to the decrease of the UbqC-E2.

Other proteases of unknown function are up-regulated in mid-logarithmic phase of growth and development of *L*. *amazonensis* promastigotes: two mitochondrial protein peptidase variants, a metal-dependent aminoexopeptidase of the M17 family and two variants of a mitochondrial processing peptidase of the M16 family that present similar MW and pI analogous to the theoretical value (Table 3). Last, the calpain LmxM.20.1280 is down-regulated in late-logarithmic phase promastigotes, whereas the paralog (LinJ.20.1230) was found to be up-regulated in *L*. *infantum* metacyclic promastigotes [33]. The physiological meaning of all these changes in proteasome components and proteases of unknown function is not known so far, although those changes at a mid time point of promastigote differentiaton suggest that they are involved in degradation of self proteins during this process.

#### Signaling

The protein kinase A (PKA) of *L*. *amazonensis* was described to be involved in autophagy [38, 39], which is a critical process in promastigote differentiation. This is in agreement with 24-fold increase of the regulatory subunit (rPKA) in stationary phase promastigotes of this species (Table 1). The rPKA is also up-regulated at the transcript level at the same growth phase of *L*. *infantum* promastigotes [20]. Therefore, these data point to an increase of abundance of the rPKA upon growth and differentiation of the promastigote stage, which may be critical for survival.

The receptor of the activated protein kinase C (RACK) of *Leishmania* spp. (LACK) is up-regulated in early-logarithmic phase promastigotes in *L*. *amazonensis* (Table 1). Constitutive expression throughout growth of *L*. *infantum* promastigotes has been reported [40]. The motile stage of *Crithidia fasciculata*, a monogenetic trypanosomatid that does not infect mammals in its life cycle, also increases the levels of the RACK orthologue CACK in early logarithmic phase [41] like *L*. *amazonensis*, according to 2DE-MALDI-TOF/TOF results obtained by the same procedure. The LACK antigen is able to protect partially against canine leishmaniasis when the encoding gene is administered in a mammalian expression plasmid vector either containing antibiotic resistance genes or alternative selection markers [42]. This protein is located in the particulate fraction of the cytoplasm near the plasma membrane and it is up-regulated in *L*. *infantum* amastigotes [40]. RACK proteins belong to the WD40 repeat family. In particular, LACK is able to bind sequences present in certain proteins involved in DNA replication and RNA synthesis and the β chain of the MHC-II. RACKs are able to translocate PKCs to the required intracellular locations [24] in order to participate in certain signal transduction pathways that have been characterized in mammalian cells [25]. The current knowledge on signal transduction pathways is still reduced in trypanosomatids. However, progress has been made with particular proteins. For example, at least one of the rPKA functions could be determined (autophagy, involved in metacyclogenesis) and it is known that LACK is a good vaccine candidate [42]. The signaling proteins 14-3-3 and putative protein phosphatase in Table 1 are more abundant in early logarithmic phase. The 14-3-3 protein is also involved in protein folding. As mentioned above, antiapoptotic function able to prolong the lifespan of the infected macrophage has been also described [35].

#### Catabolism and biosynthesis of surface molecules

**Fig 3:**
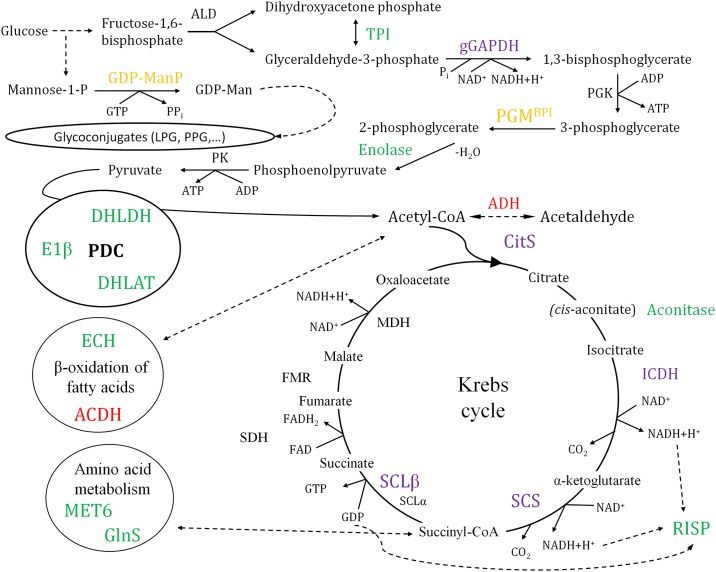
Differential abundance of proteins involved in *L*. *amazonensis* metabolism. Constitutively expressed and differentially regulated proteins that participate in glycolysis, the Krebs cycle, amino acid metabolism and β-oxidation of fatty acids are depicted. Colour legend for up-regulation: green, day 2 (lag/early log); blue, day 3 (log); orange, day 5 (late log); red, day 7 (stat). The constitutively expressed proteins experimentally detected throughout the promastigote growth curve are highlighted in violet. Abbreviations not detailed in the text: ALD, fructose-1,6-bisphosphate aldolase; ECH, enoyl-CoA hydratase/isomerase; FMR, fumarase; MET6, methyltetrahydropteroyltriglutamate-homocysteine methyltransferase; MDH, malate dehydrogenase; PGK, phosphoglycerate kinase; PK, pyruvate kinase; SDH, succinate dehydrogenase.

Glycolysis is less active in amastigotes than in promastigotes [43]. The opposite has been observed for β-oxidation of fatty acids. Most glycolytic enzymes are compartmentalized in the glycosome and some of them have been considered as potential drug targets [44–46]. Thiol groups of glycolytic enzymes are modified under nitrosative stress in *Escherichia coli* and *Mycobacterium tuberculosis*, leading to an appropriate conformational state of the protein that somehow allows for resistance [47, 48]. These findings suggest an interesting hypothetical association between steady-state levels of glycolytic enzymes in the appropriate conformational state and resistance to NO. *M*. *tuberculosis* and certain invasive *E*. *coli* strains are facultative intracellular pathogens, whereas the amastigote of *Leishmania* spp. is an obligate intracellular stage. Promastigotes are also able to survive in the presence of NO. On these grounds, we propose a hypothetical association between differential abundance of glycolytic enzymes and resistance to nitric oxide (see discussion about nitric oxide resistance below). However, this has not been experimentally tested. The triose phosphate isomerase (TPI), the enolase and the bisphosphoglycerate-independent phosphoglycerate mutase (PGM^BPI^) are differentially regulated in *L*. *amazonensis* promastigotes (Table 1), whereas the glyceraldehyde-3-phosphate dehydrogenase (gGAPDH) is constitutively expressed (Table 2). Constitutive expression of the gGAPDH was also described in the differentiation process of *L*. *infantum* promastigotes to amastigotes [28]. The abundance of the TPI, a potential vaccine candidate [49], decreases in mid-logarithmic phase promastigotes, whereas the PGM^BPI^ is up-regulated at late-logarithmic phase. The PGM^BPI^ was reported to be down-regulated in *L*. *infantum* and *L*. *donovani* amastigotes [20, 23, 24, 50] at the transcript and protein levels, respectively. Additionally, this protein is considered a promising drug target candidate [51]. Differential expression of the TPI and the PGM^BPI^ has not been detected across promastigote growth and differentiation of these visceral leishmaniasis-causative species in other studies. The enolase is down-regulated at the final stages of growth and differentiation of *L*. *amazonensis* (late-logarithmic phase) (Fig 3), which is analogous at the transcript level (over-expressed in metacyclics) in the case of *L*. *infantum* [33]. This protein is down-regulated in the amastigote stage of *L*. *mexicana*, *L*. *donovani* and *L*. *infantum* [21, 23, 24, 33]. The expression profile in *L*. *infantum* stationary phase metacyclic promastigotes is comparable at the transcript level [33].

Three components of the pyruvate dehydrogenase complex (PDC), namely the E1β subunit, dihydrolipoamide dehydrogenase (DHLDH) and dihydrolipoamide acetyltransferase (DHLAT), are significantly up-regulated in early-logarithmic phase promastigotes (Fig 3, Table 1). The aconitase is the only differentially abundant enzyme of the Krebs cycle found. This protein is over-expressed at the beginning of growth and differentiation of promastigotes (in early-logarithmic phase), like the PDC components mentioned above. On the opposite, the citrate synthase (CitS), isocitrate dehydrogenase (ICDH), succinyl-CoA synthetase α subunit (SCS) and GDP-forming succinyl-CoA ligase β chain (SCL) are constitutively expressed (Fig 3, Table 2). The Rieske iron sulfur precursor (RISP) is a protein involved in the electron transport chain. RISP abundance decreases in late-logarithmic phase promastigotes, when growth and differentiation are at an advanced stage. Krebs cycle and iron-sulfur cluster-dependent enzymes have been associated to susceptibility to NO by growth inhibition depending on the redox state of sulfhydryl groups in pathogenic bacteria [52, 53].

In summary, the differentially abundant glycolytic enzymes TPI and enolase, the DHLDH and DHLAT components of the PDC and the electron transport chain protein RISP are down-regulated in the final stages of growth and differentiation of promastigotes, except for the increase of PGM^BPI^. These changes suggest higher demand of energy during the first stages of promastigote development and might be somehow associated to nitric oxide resistance, as proposed above. On the opposite, the acyl-CoA dehydrogenase, involved in β-oxidation of fatty acids, is up-regulated in stationary phase promastigotes (Fig 3, Table 1), suggesting alternation in the energy source at this stage.

The GDP-mannose pyrophosphorylase (GDP-MP) is up-regulated in late-logarithmic phase promastigotes, which is in agreement with a previous observation in *L*. *infantum* promastigotes by the same approach [14]. The GDP-MP is essential for virulence of promastigotes of *L*. *mexicana* [54], a species closely related to *L*. *amazonensis*. This enzyme is involved in biosynthesis of the lipophosphoglycan (LPG), the glycosylinositol phospholipids (GIPLs) and other glycoconjugates characteristic of the surface of these parasites. The up-regulation of the GDP-MP in late-logarithmic phase promastigotes suggests that higher amounts of these surface molecules are synthesized when the end of growth and differentiation of promastigotes approaches.

### Up-regulation of proteins involved in resistance and survival at the last stages of growth and development of *L*. *amazonensis* promastigotes

#### Trypanothione system

**Fig 4:**
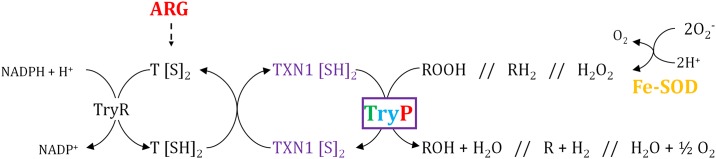
Differential abundance of proteins involved in redox homeostasis. The FE-SOD participates in ROS processing providing hydrogen peroxide, which is then reduced by the TryP with the assistance of the TXN1, trypanithione and TryR. TryP differential expression pattern is complex through the promastigote growth curve of *L*. *amazonensis*. A light TryP variant is up-regulated in stationary phase promastigotes. A heavier TryP variant is down-regulated at day 3 and another one up-regulated simultaneously. Finally, a fourth variant is constitutively expressed during promastigote growth. Colour legend for up-regulation: green, day 2 (lag/early log); blue, day 3 (log); orange, day 5 (late log); red, day 7 (stat). The constitutively expressed proteins experimentally detected throughout the promastigote growth curve are highlighted in violet.

A processed protein variant (12.9 KDa, pI8.5) of the tryparedoxin peroxidase (TryP) is constitutively expressed throughout the growth curve of *L*. *amazonensis* promastigotes, whereas two heavier variants of estimated experimental MW and pI close to the theoretical values (22.0 KDa, pI6.0) are differentially abundant (Fig 4, Tables 1 and 3). One of the TryP heavy variants is up-regulated at day 3, whereas the other one is down-regulated at the same time point. The TryP light variant is highly up-regulated (>30-fold) in stationary phase promastigotes (day 7). In the case of *L*. *infantum*, the TryP decreases in amastigotes compared to promastigotes [28] but there are no available data about relative abundance during growth and differentiation process of amastigotes to date. The complex expression profiles of different TryP variants in *L*. *amazonensis* promastigotes (Fig 4) has not been found in Old World *Leishmania* species such as *L*. *major* or *L*. *infantum* so far and the enzyme was reported to be constitutively expressed in both species [20, 55], whereas the non-pathogenic trypanosomatid *C*. *fasciculata* differentially regulates the TryP across growth and differentiation of the motile choanomastigote stage [41]. In addition to cellular detoxification of reactive oxygen species, the TryP has been involved in other processes such signaling proliferation and differentiation [56]. The complex expression pattern of different variants found in *L*. *amazonensis* promastigotes (Fig 4) suggests that the TryP is especially important throughout the growth and differentiation process studied herein.

The iron superoxide dismutase (Fe-SOD) is over-expressed in *L*. *amazonensis* and in *C*. *fasciculata*. This protein initiates elimination of the superoxide anion by conversion to hydrogen peroxide. The Fe-SOD is up-regulated in *L*. *amazonensis* late logarithmic phase promastigotes (day 5). The TryP is able to reduce this substrate thanks to the assistance of the tryparedoxin (TXN1), the trypanothione (T[S]_2_/T[SH]_2_) and the NADPH-dependent enzyme trypanothione reductase (TryR). The TryR has not been identified between the selected spots of this analysis and the TXN1 is among the constitutively expressed ones (Table 2).

#### Arginase

**Fig 5:**
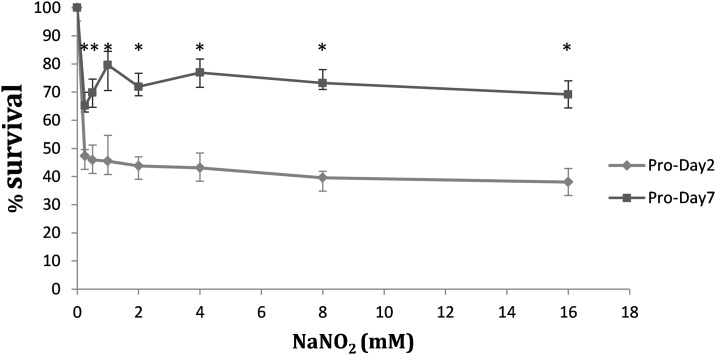
Resistance to NO of logarithmic and stationary phase promastigotes of *L*. *amazonensis*. The MTT assay was performed to compare the ability to resist increasing concentrations of NaNO_2_ by logarithmic and stationary phase promastigotes of *L*. *amazonensis*. Three biological replicates of the experiment were performed and the differences, contrasted by the paired t-test, were significant at all NaNO_2_ concentrations tested (p*<0.001).

The arginase is a manganese-dependent enzyme that participates in the urea cycle by hydrolyzing L-arginine into urea and ornithine. These products are able to induce the alternative macrophage activation pathway [57]. The ARG of the host phagocytic cell competes with the inducible nitric oxide synthase (iNOS) for the substrate L-arginine [58]. For these reasons, parasite clearance is more effective when low levels of host ARG are expressed. The ARG is also annotated in the genomes of the *Leishmania* species, thus contributing to decrease the levels of NO synthesis by the iNOS of the host cell through substrate competence. This enzyme is essential for infectivity, proliferation and virulence of the parasite [59, 60]. 2DE-MALDI-TOF/TOF analysis has revealed high ARG levels (29-fold) in *L*. *amazonensis* stationary phase promastigotes. *L*. *infantum* promastigotes overexpress the ARG in amastigotes with respect to stationary phase promastigotes [28]. A possible explanation for these findings combined is preparation in advance for differentiation to the amastigote stage and survival within the phagolysosome of the host cell. This statement is called pre-adaptation hypothesis and has been previously supported [20, 61–63]. The MTT test has confirmed that *L*. *amazonensis* stationary phase promastigotes are more resistant to NO than logarithmic phase promastigotes (Fig 5). Therefore, NO resistance increases in the ongoing of growth and development of promastigotes in culture.

Ornithine is an essential precursor in polyamine biosynthesis [54, 55]. This pathway is essential for the biosynthesis of trypanothione because this molecule consists of two glutathione residues coupled through spermidine. Consequently, arginase up-regulation might be indirectly related to the TryP differential expression profile found (Fig 4).

#### Synopsis of differentially abundant proteins involved in survival and resistance

Decreased abundance of a variant of the STI1 chaperone simultaneous to the increase of the levels of the ARG, HSP70, TryP, Fe-SOD (stress-resistance proteins) and rPKA (indicative of differentiation) has been found when growth and differentiation of *L*. *amazonensis* promastigotes is complete. These changes, especially ARG up-regulation, may be linked to the increased ability of differentiated promastigotes to resist NO with respect to the non-differentiated, which is in agreement with the pre-adaptation hypothesis towards survival within the host phagocyte and may explain unsuccessful clearance by the immune system. In addition, higher levels of the GDP-ManP have been observed at an advanced growth phase, which probably leads to increased amounts of glycoconjugates in differentiated promastigotes.

## Conclusions

2DE-MS/MS has revealed that promastigotes of the New World species *L*. *amazonensis*, responsible for ACL, up-regulate proteins involved in survival and resistance throughout growth and development in culture: the TryP and the Fe-SOD, involved in defense against oxidative damage; the ARG, involved in nitrosative stress resistance within the phagocytic host cell, which is up-regulated in stationary phase promastigotes, thus supporting the pre-adaptative hypothesis; the immunostimulatory protein HSP 70, involved in protein folding; and the rPKA, involved in signaling and autophagy, thus related with metacyclogenesis. The LACK antigen is down-regulated throughout growth and simultaneous development of promastigotes in this species causative of ACL, whereas it was reported to be constitutively expressed in the VL agent *L*. *infantum*. The down-regulation of the STI1 and the up-regulation of proteins involved in resistance and survival (ARG, HSP70, Fe-SOD, TryP and rPKA) in fully grown and differentiated promastigotes suggest the pre-adaptative hypothesis, also proposed for other *Leishmania* species.

## Supporting Information

S1 Fig Scatter plot of differential protein abundance in *L*. *amazonensis* promastigotes. (PPTX)

S1 Table Spot density values. (TXT)

## References

[1] Convit J, Lapenta P. Sobre un caso de leishmaniasis diseminada. Rev Pat Clin. 1946;17:153–8.

[2] WHO. Report of a Meeting of the WHO Expert Committee on the Control of Leishmaniases. Geneva: 2010.

[3] WHO. Leishmaniases. Epidemiological Report of the Americas 2015 [cited 2015]. Available from: http://www.paho.org/hq/index.php?option=com_docman&task=doc_download&Itemid=270&gid=31142&lang=en.

[4] Ivens AC, Peacock CS, Worthey EA, Murphy L, Aggarwal G, Berriman M, et al The genome of the kinetoplastid parasite, Leishmania major. Science. 2005;309(5733):436–42. Epub 2005/07/16. 309/5733/436 [pii] 10.1126/science.1112680 16020728 PMC1470643

[5] Peacock CS, Seeger K, Harris D, Murphy L, Ruiz JC, Quail MA, et al Comparative genomic analysis of three Leishmania species that cause diverse human disease. Nat Genet. 2007;39(7):839–47. Epub 2007/06/19. ng2053 [pii] 10.1038/ng2053 17572675 PMC2592530

[6] Aslett M, Aurrecoechea C, Berriman M, Brestelli J, Brunk BP, Carrington M, et al TriTrypDB: a functional genomic resource for the Trypanosomatidae. Nucleic Acids Res. 2010;38(Database issue):D457–62. 10.1093/nar/gkp851 19843604 PMC2808979

[7] de Toledo JS, Vasconcelos EJR, Ferreira TR, Cruz AK. Using Genomic Information to Understand Leishmania Biology. Open Parasitol J. 2010;4:156–66.

[8] Brobey RK, Mei FC, Cheng X, Soong L. Comparative two-dimensional gel electrophoresis maps for promastigotes of Leishmania amazonensis and Leishmania major. The Brazilian journal of infectious diseases: an official publication of the Brazilian Society of Infectious Diseases. 2006;10(1):1–6. . 16767307 10.1590/s1413-86702006000100001

[9] Brobey RK, Soong L. Establishing a liquid-phase IEF in combination with 2-DE for the analysis of Leishmania proteins. Proteomics. 2007;7(1):116–20. 10.1002/pmic.200600587 . 17124718

[10] Magalhaes RD, Duarte MC, Mattos EC, Martins VT, Lage PS, Chavez-Fumagalli MA, et al Identification of differentially expressed proteins from Leishmania amazonensis associated with the loss of virulence of the parasites. PLoS Negl Trop Dis. 2014;8(4):e2764 10.1371/journal.pntd.0002764 24699271 PMC3974679

[11] do Monte-Neto RL, Coelho AC, Raymond F, Legare D, Corbeil J, Melo MN, et al Gene expression profiling and molecular characterization of antimony resistance in Leishmania amazonensis. PLoS Negl Trop Dis. 2011;5(5):e1167 10.1371/journal.pntd.0001167 21629719 PMC3101167

[12] Dominguez M, Torano A. Leishmania immune adherence reaction in vertebrates. Parasite Immunol. 2001;23(5):259–65. Epub 2001/04/20. pim380 [pii]. . 11309136 10.1046/j.1365-3024.2001.00380.x

[13] Wessel D, Flugge UI. A method for the quantitative recovery of protein in dilute solution in the presence of detergents and lipids. Anal Biochem. 1984;138(1):141–3. . 6731838 10.1016/0003-2697(84)90782-6

[14] Alcolea PJ, Alonso A, Larraga V. Proteome profiling of Leishmania infantum promastigotes. J Eukaryot Microbiol. 2011;58(4):352–8. Epub 2011/05/17. 10.1111/j.1550-7408.2011.00549.x . 21569158

[15] Vizcaino JA, Deutsch EW, Wang R, Csordas A, Reisinger F, Rios D, et al ProteomeXchange provides globally coordinated proteomics data submission and dissemination. Nature biotechnology. 2014;32(3):223–6. 10.1038/nbt.2839 24727771 PMC3986813

[16] Mosmann T. Rapid colorimetric assay for cellular growth and survival: application to proliferation and cytotoxicity assays. Journal of immunological methods. 1983;65(1–2):55–63. . 6606682 10.1016/0022-1759(83)90303-4

[17] Bras-Goncalves R, Petitdidier E, Pagniez J, Veyrier R, Cibrelus P, Cavaleyra M, et al Identification and characterization of new Leishmania promastigote surface antigens, LaPSA-38S and LiPSA-50S, as major immunodominant excreted/secreted components of L. amazonensis and L. infantum. Infection, genetics and evolution: journal of molecular epidemiology and evolutionary genetics in infectious diseases. 2014;24:1–14. 10.1016/j.meegid.2014.02.017 . 24614507

[18] Clayton C, Shapira M. Post-transcriptional regulation of gene expression in trypanosomes and leishmanias. Mol Biochem Parasitol. 2007;156(2):93–101. 10.1016/j.molbiopara.2007.07.007 . 17765983

[19] Gupta SK, Sisodia BS, Sinha S, Hajela K, Naik S, Shasany AK, et al Proteomic approach for identification and characterization of novel immunostimulatory proteins from soluble antigens of Leishmania donovani promastigotes. Proteomics. 2007;7(5):816–23. Epub 2007/02/14. 10.1002/pmic.200600725 . 17295358

[20] Alcolea PJ, Alonso A, Gomez MJ, Moreno I, Dominguez M, Parro V, et al Transcriptomics throughout the life cycle of Leishmania infantum: high down-regulation rate in the amastigote stage. Int J Parasitol. 2010;40(13):1497–516. Epub 2010/07/27. 10.1016/j.ijpara.2010.05.013 S0020-7519(10)00252-3 [pii]. . 20654620

[21] Holzer TR, McMaster WR, Forney JD. Expression profiling by whole-genome interspecies microarray hybridization reveals differential gene expression in procyclic promastigotes, lesion-derived amastigotes, and axenic amastigotes in Leishmania mexicana. Mol Biochem Parasitol. 2006;146(2):198–218. 10.1016/j.molbiopara.2005.12.009 . 16430978

[22] Leifso K, Cohen-Freue G, Dogra N, Murray A, McMaster WR. Genomic and proteomic expression analysis of Leishmania promastigote and amastigote life stages: the Leishmania genome is constitutively expressed. Mol Biochem Parasitol. 2007;152(1):35–46. Epub 2006/12/26. S0166-6851(06)00330-6 [pii] 10.1016/j.molbiopara.2006.11.009 . 17188763

[23] Rosenzweig D, Smith D, Myler PJ, Olafson RW, Zilberstein D. Post-translational modification of cellular proteins during Leishmania donovani differentiation. Proteomics. 2008;8(9):1843–50. Epub 2008/04/10. 10.1002/pmic.200701043 . 18398879

[24] Rosenzweig D, Smith D, Opperdoes F, Stern S, Olafson RW, Zilberstein D. Retooling Leishmania metabolism: from sand fly gut to human macrophage. FASEB J. 2008;22(2):590–602. 10.1096/fj.07-9254com . 17884972

[25] Zilberstein D, Shapira M. The role of pH and temperature in the development of Leishmania parasites. Annual review of microbiology. 1994;48:449–70. 10.1146/annurev.mi.48.100194.002313 . 7826014

[26] Wiesgigl M, Clos J. The heat shock protein 90 of Leishmania donovani. Medical microbiology and immunology. 2001;190(1–2):27–31. . 11770104 10.1007/s004300100074

[27] Carrillo E, Crusat M, Nieto J, Chicharro C, Thomas Mdel C, Martinez E, et al Immunogenicity of HSP-70, KMP-11 and PFR-2 leishmanial antigens in the experimental model of canine visceral leishmaniasis. Vaccine. 2008;26(15):1902–11. Epub 2008/03/07. 10.1016/j.vaccine.2008.01.042 S0264-410X(08)00101-1 [pii]. . 18321614

[28] Lynn MA, Marr AK, McMaster WR. Differential quantitative proteomic profiling of Leishmania infantum and Leishmania mexicana density gradient separated membranous fractions. Journal of proteomics. 2013;82:179–92. 10.1016/j.jprot.2013.02.010 . 23466312

[29] Bhaskar, Mitra K, Kuldeep J, Siddiqi MI, Goyal N. The TCP1gamma subunit of Leishmania donovani forms a biologically active homo-oligomeric complex. FEBS J. 2015;282(23):4607–19. Epub 2015/09/24. 10.1111/febs.13521 . 26395202

[30] Alcolea PJ, Alonso A, Dominguez M, Parro V, Jimenez M, Molina R, et al Influence of the Microenvironment in the Transcriptome of Leishmania infantum Promastigotes: Sand Fly versus Culture. PLoS Negl Trop Dis. 2016;10(5):e0004693 Epub 2016/05/11. 10.1371/journal.pntd.0004693 PNTD-D-15-00823 [pii]. 27163123 PMC4862625

[31] Chakraborty A, Sen B, Datta R, Datta AK. Isomerase-independent chaperone function of cyclophilin ensures aggregation prevention of adenosine kinase both in vitro and under in vivo conditions. Biochemistry. 2004;43(37):11862–72. Epub 2004/09/15. 10.1021/bi049490o . 15362872

[32] Sen B, Venugopal V, Chakraborty A, Datta R, Dolai S, Banerjee R, et al Amino acid residues of Leishmania donovani cyclophilin key to interaction with its adenosine kinase: biological implications. Biochemistry. 2007;46(26):7832–43. Epub 2007/06/08. 10.1021/bi602625h . 17552497

[33] Alcolea PJ, Alonso A, Sanchez-Gorostiaga A, Moreno-Paz M, Gomez MJ, Ramos I, et al Genome-wide analysis reveals increased levels of transcripts related with infectivity in peanut lectin non-agglutinated promastigotes of Leishmania infantum. Genomics. 2009;93(6):551–64. Epub 2009/05/16. 10.1016/j.ygeno.2009.01.007 S0888-7543(09)00025-1 [pii]. . 19442635

[34] Yano M, Mori S, Niwa Y, Inoue M, Kido H. Intrinsic nucleoside diphosphate kinase-like activity as a novel function of 14-3-3 proteins. FEBS letters. 1997;419(2–3):244–8. . 9428643 10.1016/s0014-5793(97)01469-5

[35] Silverman JM, Chan SK, Robinson DP, Dwyer DM, Nandan D, Foster LJ, et al Proteomic analysis of the secretome of Leishmania donovani. Genome Biol. 2008;9(2):R35 Epub 2008/02/20. 10.1186/gb-2008-9-2-r35 gb-2008-9-2-r35 [pii]. 18282296 PMC2374696

[36] Alcolea PJ. Análisis de los perfiles de expresión génica en los procesos de diferenciación de Leishmania infantum mediante microarrays de ADN. E-Prints Complutense: Universidad Complutense de Madrid; 2011.

[37] da Fonseca Pires S, Fialho LC Jr., Silva SO, Melo MN, de Souza CC, Tafuri WL, et al Identification of virulence factors in Leishmania infantum strains by a proteomic approach. J Proteome Res. 2014;13(4):1860–72. Epub 2014/03/13. 10.1021/pr400923g . 24617796

[38] Genestra M, Cysne-Finkelstein L, Leon L. Protein kinase A activity is associated with metacyclogenesis in Leishmania amazonensis. Cell biochemistry and function. 2004;22(5):315–20. 10.1002/cbf.1107 . 15338471

[39] Bhattacharya A, Biswas A, Das PK. Identification of a protein kinase A regulatory subunit from Leishmania having importance in metacyclogenesis through induction of autophagy. Mol Microbiol. 2012;83(3):548–64. Epub 2011/12/16. 10.1111/j.1365-2958.2011.07950.x . 22168343

[40] Gonzalez-Aseguinolaza G, Taladriz S, Marquet A, Larraga V. Molecular cloning, cell localization and binding affinity to DNA replication proteins of the p36/LACK protective antigen from Leishmania infantum. Eur J Biochem. 1999;259(3):909–16. Epub 1999/03/27. . 10092881 10.1046/j.1432-1327.1999.00122.x

[41] Alcolea PJ, Alonso A, Garcia-Tabares F, Torano A, Larraga V. An Insight into the proteome of Crithidia fasciculata choanomastigotes as a comparative approach to axenic growth, peanut lectin agglutination and differentiation of Leishmania spp. promastigotes. PLoS One. 2014;9(12):e113837 Epub 2014/12/17. 10.1371/journal.pone.0113837 PONE-D-14-29467 [pii]. 25503511 PMC4263474

[42] Ramos I, Alonso A, Peris A, Marcen JM, Abengozar MA, Alcolea PJ, et al Antibiotic resistance free plasmid DNA expressing LACK protein leads towards a protective Th1 response against Leishmania infantum infection. Vaccine. 2009;27(48):6695–703. Epub 2009/09/15. 10.1016/j.vaccine.2009.08.091 S0264-410X(09)01300-0 [pii]. . 19747996

[43] Coombs GH, Craft JA, Hart DT. A comparative study of Leishmania mexicana amastigotes and promastigotes. Enzyme activities and subcellular locations. Mol Biochem Parasitol. 1982;5(3):199–211. . 6211617 10.1016/0166-6851(82)90021-4

[44] Barrett MP, Mottram JC, Coombs GH. Recent advances in identifying and validating drug targets in trypanosomes and leishmanias. Trends Microbiol. 1999;7(2):82–8. Epub 1999/03/19. S0966-842X(98)01433-4 [pii]. . 10081086 10.1016/s0966-842x(98)01433-4

[45] Croft SL, Yardley V. Chemotherapy of leishmaniasis. Curr Pharm Des. 2002;8(4):319–42. Epub 2002/02/28. . 11860369 10.2174/1381612023396258

[46] Verlinde CL, Hannaert V, Blonski C, Willson M, Perie JJ, Fothergill-Gilmore LA, et al Glycolysis as a target for the design of new anti-trypanosome drugs. Drug Resist Updat. 2001;4(1):50–65. Epub 2001/08/22. S1368-7646(00)90177-8 [pii] . 11512153 10.1054/drup.2000.0177

[47] Brandes N, Rinck A, Leichert LI, Jakob U. Nitrosative stress treatment of E. coli targets distinct set of thiol-containing proteins. Mol Microbiol. 2007;66(4):901–14. 10.1111/j.1365-2958.2007.05964.x 17919278 PMC2794660

[48] Rhee KY, Erdjument-Bromage H, Tempst P, Nathan CF. S-nitroso proteome of Mycobacterium tuberculosis: Enzymes of intermediary metabolism and antioxidant defense. Proc Natl Acad Sci U S A. 2005;102(2):467–72. 10.1073/pnas.0406133102 15626759 PMC544291

[49] Kushawaha PK, Gupta R, Tripathi CD, Khare P, Jaiswal AK, Sundar S, et al Leishmania donovani triose phosphate isomerase: a potential vaccine target against visceral leishmaniasis. PLoS One. 2012;7(9):e45766 Epub 2012/10/11. 10.1371/journal.pone.0045766 PONE-D-12-09376 [pii]. 23049855 PMC3454378

[50] Alcolea PJ, Alonso A, Gomez MJ, Sanchez-Gorostiaga A, Moreno-Paz M, Gonzalez-Pastor E, et al Temperature increase prevails over acidification in gene expression modulation of amastigote differentiation in Leishmania infantum. BMC Genomics. 2010;11:31 Epub 2010/01/16. 10.1186/1471-2164-11-31 1471-2164-11-31 [pii]. 20074347 PMC2845110

[51] Guerra DG, Vertommen D, Fothergill-Gilmore LA, Opperdoes FR, Michels PA. Characterization of the cofactor-independent phosphoglycerate mutase from Leishmania mexicana mexicana. Histidines that coordinate the two metal ions in the active site show different susceptibilities to irreversible chemical modification. Eur J Biochem. 2004;271(9):1798–810. Epub 2004/04/21. 10.1111/j.1432-1033.2004.04097.x EJB4097 [pii]. . 15096219

[52] Brown GC, McBride AG, Fox EJ, McNaught KS, Borutaite V. Nitric oxide and oxygen metabolism. Biochemical Society transactions. 1997;25(3):901–4. . 9388569 10.1042/bst0250901

[53] Richardson AR, Payne EC, Younger N, Karlinsey JE, Thomas VC, Becker LA, et al Multiple targets of nitric oxide in the tricarboxylic acid cycle of Salmonella enterica serovar typhimurium. Cell host & microbe. 2011;10(1):33–43. 10.1016/j.chom.2011.06.004 21767810 PMC3142370

[54] Davis AJ, Perugini MA, Smith BJ, Stewart JD, Ilg T, Hodder AN, et al Properties of GDP-mannose pyrophosphorylase, a critical enzyme and drug target in Leishmania mexicana. J Biol Chem. 2004;279(13):12462–8. Epub 2004/01/14. 10.1074/jbc.M312365200 M312365200 [pii]. . 14718535

[55] Tetaud E, Fairlamb AH. Cloning, expression and reconstitution of the trypanothione-dependent peroxidase system of Crithidia fasciculata. Mol Biochem Parasitol. 1998;96(1–2):111–23. . 9851611 10.1016/s0166-6851(98)00120-0

[56] Castro H, Sousa C, Santos M, Cordeiro-da-Silva A, Flohe L, Tomas AM. Complementary antioxidant defense by cytoplasmic and mitochondrial peroxiredoxins in Leishmania infantum. Free Radic Biol Med. 2002;33(11):1552–62. Epub 2002/11/26. S0891584902010894 [pii]. . 12446213 10.1016/s0891-5849(02)01089-4

[57] Gordon S. Alternative activation of macrophages. Nature reviews Immunology. 2003;3(1):23–35. 10.1038/nri978 . 12511873

[58] Goerdt S, Politz O, Schledzewski K, Birk R, Gratchev A, Guillot P, et al Alternative versus classical activation of macrophages. Pathobiology. 1999;67(5–6):222–6. . 10725788 10.1159/000028096

[59] Gaur U, Roberts SC, Dalvi RP, Corraliza I, Ullman B, Wilson ME. An effect of parasite-encoded arginase on the outcome of murine cutaneous leishmaniasis. Journal of immunology. 2007;179(12):8446–53. . 18056391 10.4049/jimmunol.179.12.8446

[60] Muleme HM, Reguera RM, Berard A, Azinwi R, Jia P, Okwor IB, et al Infection with arginase-deficient Leishmania major reveals a parasite number-dependent and cytokine-independent regulation of host cellular arginase activity and disease pathogenesis. Journal of immunology. 2009;183(12):8068–76. 10.4049/jimmunol.0803979 19923451 PMC2800308

[61] Alcolea PJ, Alonso A, Gomez MJ, Postigo M, Molina R, Jimenez M, et al Stage-specific differential gene expression in Leishmania infantum: from the foregut of Phlebotomus perniciosus to the human phagocyte. BMC Genomics. 2014;15:849 Epub 2014/10/05. 10.1186/1471-2164-15-849 1471-2164-15-849 [pii]. 25281593 PMC4203910

[62] Bates PA. Housekeeping by Leishmania. Trends Parasitol. 2006;22(10):447–8. Epub 2006/08/15. S1471-4922(06)00204-2 [pii] 10.1016/j.pt.2006.08.003 16905359 PMC2679205

[63] Depledge DP, Evans KJ, Ivens AC, Aziz N, Maroof A, Kaye PM, et al Comparative expression profiling of Leishmania: modulation in gene expression between species and in different host genetic backgrounds. PLoS Negl Trop Dis. 2009;3(7):e476 Epub 2009/07/08. 10.1371/journal.pntd.0000476 19582145 PMC2701600

