# Supplementary material for: Increased Abundance of Proteins Involved in Resistance to Oxidative and Nitrosative Stress at the Last Stages of Growth and Development of Leishmania amazonensis Promastigotes Revealed by Proteome Analysis
Source: PLoS One. 2016 Oct 24;11(10):e0164344. doi: 10.1371/journal.pone.0164344 (PMC5077082; doi:10.1371/journal.pone.0164344)
Supplement: S1 Fig — (PPTX) [file pone.0164344.s001.pptx]

## Slide 1
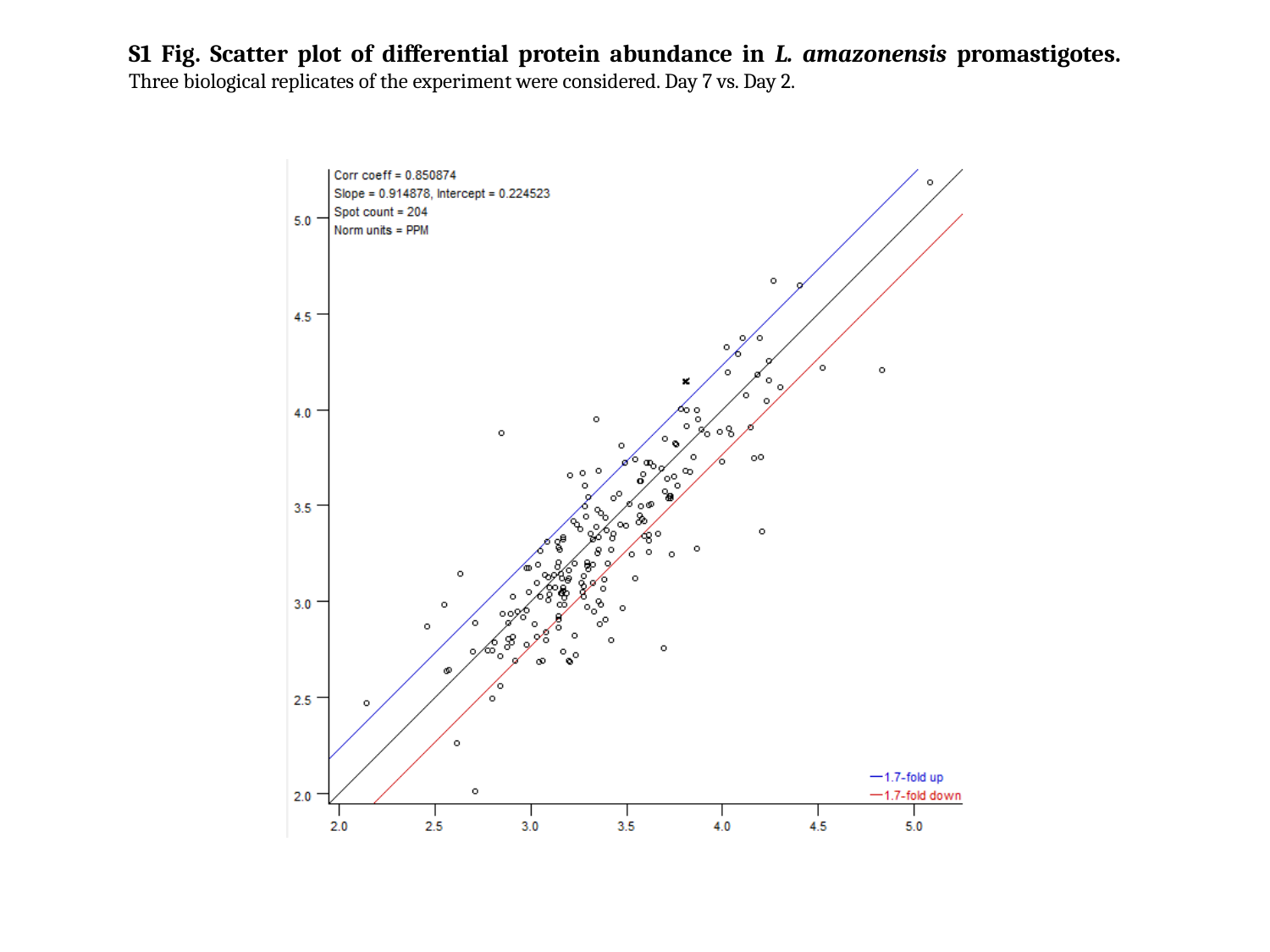

S1 Fig. Scatter plot of differential protein abundance in L. amazonensis promastigotes. Three biological replicates of the experiment were considered. Day 7 vs. Day 2.
